# Taxonomic Revision of the Relationship Between *Coproptilia* and *Nosphistica* (Lepidoptera: Lecithoceridae) with Descriptions of Two New Species and a New Record from China

**DOI:** 10.3390/ani15030426

**Published:** 2025-02-04

**Authors:** Haotian Li, Shuai Yu

**Affiliations:** College of Agriculture and Biology, Liaocheng University, Liaocheng 252059, China; lihaotian@lcu.edu.cn

**Keywords:** *Coproptilia*, *Nosphistica*, taxonomy, new species, new record

## Abstract

The family Lecithoceridae represents one of the most diverse yet understudied groups within Lepidoptera, with numerous unresolved taxonomic issues requiring urgent attention. Among these, the genus *Coproptilia* Snellen, a small genus within Lecithoceridae, has traditionally been distinguished from its closely related genus, *Nosphistica* Meyrick, primarily by the presence of vein R_1_ in the forewing. However, *Nosphistica* exhibits considerable interspecific variation in wing venation, complicating clear differentiation between these genera. In this study, we conducted a molecular phylogenetic analysis of Lecithoceridae based on a concatenated dataset of 5350 bp, comprising one mitochondrial marker and six nuclear markers from 28 Lecithoceridae individuals. The result shows that (1) the topological results of both Maximum Likelihood (ML) and Bayesian Inference (BI) trees show the *Coproptilia* branch embedded within the *Nosphistica* branch, so *Nosphistica* Meyrick should be synonymized with *Coproptilia* Snellen. (2) Two new species are described from China: *Coproptilia uniformis* Yu, sp. nov. and *C. funiuensis* Yu, sp. nov. (3) *Coproptilia tawiensis* Park, 2009, is newly recorded from China, with the first description of its female. Finally, we integrated molecular and morphological evidence to examine the taxonomic relationship between *Coproptilia* and *Nosphistica*. Additional molecular data will be needed to further clarify the subfamily affiliation of *Coproptilia* sensu nov.

## Simple Summary

The family Lecithoceridae is one of the most diverse and understudied groups within Lepidoptera. Within this family, the taxonomic relationship between the genus *Coproptilia* Snellen and its closely related genus *Nosphistica* Meyrick has remained confused due to morphological variations. In this study, we integrated molecular and morphological methods to discuss the taxonomic relationship between *Coproptilia* and *Nosphistica*. Our results indicate that *Nosphistica* should be synonymized with *Coproptilia*. Additionally, we report two new species and one new record of *Coproptilia* from China.

## 1. Introduction

The family Lecithoceridae is the sixth largest within the superfamily Gelechioidea, encompassing more than 1430 species [1,2]. This family has a wide distribution across the Oriental, Ethiopian, Australian, and Palaearctic regions. Members of Lecithoceridae are typically characterized by the antennae, which are as long as or longer than the forewing, and by the gnathos in the male genitalia, which usually features a median process that is consistently downturned, except in the subfamily Crocanthinae. Despite its remarkable diversity, Lecithoceridae has received very limited scientific attention. In certain regions, such as southern China, this family is a dominant group; however, the lack of research interest may persist due to the group’s minimal economic significance and the shortage of specialists in this field. Interestingly, Lecithoceridae larvae have been reported to feed on non-living materials and organisms [3,4,5,6], highlighting their potentially significant role in environmental ecosystems.

*Coproptilia* is a small genus of Lecithoceridae, first established by Snellen, 1903, with *C. glebicolorella* from Indonesia designated as the type species [7]. Wu later described the second species, *C. diona,* from China [8], followed by Park’s description of a third species, *C. tawiensis,* from the Philippines [9]. Currently, only three species of the genus *Coproptilia* are known worldwide. Wu initially classified the genus within the subfamily Torodorinae [10], but Park et al. excluded it from Torodorinae in their monograph on the subfamily [2].

*Nosphistica* was established by Meyrick, 1911 [11], and currently comprises 22 species. The species within this genus are geographically restricted to the Oriental region, with 17 species recorded in China. Wu classified *Nosphistica* within the subfamily Lecithocerinae [10], but Park suggested that the genus represents an intermediate group between Lecithocerinae and Torodorinae [12]. Furthermore, Park synonymized *Philoptila* Meyrick, 1918, with *Nosphistica* [12].

The genera *Coproptilia* and *Nosphistica* share several morphological characteristics, including the ventrally ciliate flagellum of the male antenna, a darkened area between vein 3A and the dorsum of the hindwing, and the costal bar of the valva, which is conspicuously free at the base but fused to the valva distally. The only distinguishing feature of *Coproptilia* is the presence of the R_1_ vein in the forewing, which is absent in *Nosphistica*, according to current criteria [12,13]. However, it is questionable whether the presence of the R_1_ vein alone is sufficient as the sole criterion for distinguishing these two genera, considering the significant intra-generic variation in wing vein patterns.

To date, the taxonomy of Lecithoceridae has primarily been based on morphological characteristics, with relatively few studies employing molecular methods. Sterling et al. conducted the first phylogenetic analysis of Lecithoceridae based on multiple molecular markers, incorporating 17 multi-genetic exemplars [14]. However, molecular data for the family remain limited, with only one dataset currently available for *Nosphistica*. Therefore, a combined approach integrating both molecular data and morphological characteristics is crucial and meaningful to resolve the relationship between *Coproptilia* and *Nosphistica*.

The primary objectives of this study are twofold: first, to clarify the taxonomic relationship between the genera *Coproptilia* and *Nosphistica*, and second, to describe two new species and report one new record from China.

## 2. Materials and Methods

The examined specimens were collected using GYZ 450 W high-pressure mercury lamps (Yaming, Shanghai, China). Morphological terminology used in the descriptions follows Gozmány [3]. Wingspan measurements were taken from the tips of the left and right forewings of fully spread specimens. Wing venation slides and genitalia slides were prepared following the methods introduced by Li [15]. Photographs of adults were captured using an M205A stereomicroscope, and genitalia photographs were taken using a DM750 microscope with Leica Application Suite software version 4.6 (Leica, Wetzlar, Germany). All images were processed with Photoshop CC (Adobe, San Jose, CA, USA). The type series of the new species are deposited at the Insect Collection of Nankai University (NKU), Tianjin, China (NKU), and at Liaocheng University (LCU), Liaocheng, China.

In this study, a total of 11 Lecithoceridae specimens were independently collected for molecular analysis. These included two specimens of *Spatulignatha olaxana* (Vouchers: LCU054 and LCU058), one of *Frisilia cornualis* (Voucher: LCU063), one of *Tegenocharis tenebrans* (Voucher: LCU331), one of *Nosphistica eucalla* (Voucher: YUS007), one of *Nosphistica paramecola* (Voucher: YUS010), one of *Nosphistica grandiunca* (Voucher: YUS011), one of *Synesarga breviclavata* (Voucher: YUS012), and three of *Coproptilia tawinensis* (Vouchers: YUS033, YUS038, and LCU369). Genomic DNA was extracted from the legs or partial bodies of dried specimens using the Magnetic Animal Tissue Genomic DNA Kit (Tiangen Biotech, Beijing, China).

One mitochondrial marker (cytochrome oxidase subunit 1 [COI]) and six nuclear markers (carbamoyl phosphate synthetase domain protein [CAD], elongation factor 1 alpha [EF-1α], glyceraldhyde-3-phosphate dehydrogenase [GAPDH], cytosolic malate dehydrogenase [MDH], ribosomal protein S5 [RpS5] gene, and wingless) were amplified using a polymerase chain reaction (PCR). The primers used were sourced from previous studies [16,17,18,19]. When the published primers failed to amplify sequences, newly designed primers were used to obtain shorter fragments of the target regions in this study (Table 1). DNA amplification and sequencing protocols primarily followed those described by Wahlberg and Wheat [19]. The purified PCR products were directly sequenced using Sanger sequencing by Qingke Biotech (Beijing, China).

To construct a more comprehensive phylogenetic tree for Lecithoceridae, a 5350 bp dataset was downloaded from GenBank, which includes all available mixed COI and 6 nuclear gene sequences of 17 Lecithoceridae individuals [1,20,21]. This dataset included 1475 bp of COI, 850 bp of CAD, 691 bp of GAPDH, 925 bp of EF-1α, 600 bp of RpS5, 400 bp of wingless, and 407 bp of MDH (Table S1).

The sequences were manually edited using BioEdit v.7.2.5 [22] and analyzed with MEGA ⅹ software [23]. Each gene (COI, CAD, GAPDH, EF-1α, RpS5, wingless, and MDH) was independently aligned and subsequently concatenated into a dataset with a total length of 5350 bp using PhyloSuite v1.2.2 [24]. Phylogenetic reconstructions of Lecithoceridae species were performed based on this concatenated dataset using Maximum Likelihood (ML) in IQ-TREE [25] and Bayesian Inference (BI) in MrBayes 3.2 [26]. The best-fit model of sequence evolution for each locus alignment was selected using the Akaike Information Criterion (AIC) in PartitionFinder v2 [27]. The selected models were as follows: GTR + I + G for COI, GTR + G for CAD, HKY + G for MDH, and SYM + I + G for wingless, RpS5, EF-1α, and GAPDH. Bootstrap support values were calculated using a rapid bootstrapping algorithm with 1000 replicates in the ML analysis. For the BI analysis, four Markov chain Monte Carlo (MCMC) runs with four chains were performed for 20,000,000 generations, sampling every 1000 trees and discarding the first 20% as burn ins.

## 3. Results

### 3.1. Molecular Analysis Results

We obtained a total of 3438 bp of sequences for our specimen, which included 647 bp of COI for eleven individuals, 759 bp of CAD for four individuals, 345 bp of EF-1α for eleven individuals, 521 bp of GAPDH for nine individuals, 335 bp of MDH for three individuals, 502 bp of RpS5 for ten individuals, and 329 bp of wingless for seven individuals. These gene sequences generated in this study have been deposited in GenBank under the following accession numbers: PQ820469-PQ820479 (COI), PQ819874-PQ8198777 (CAD), PQ819878-PQ819888 (EF-1α), PQ819889-PQ819897 (GAPDH), PQ819898-PQ819900 (MDH) PQ819901-PQ819909 (RpS5), and PQ819911-PQ819917 (wingless) (Table S1).

**Figure 1:**
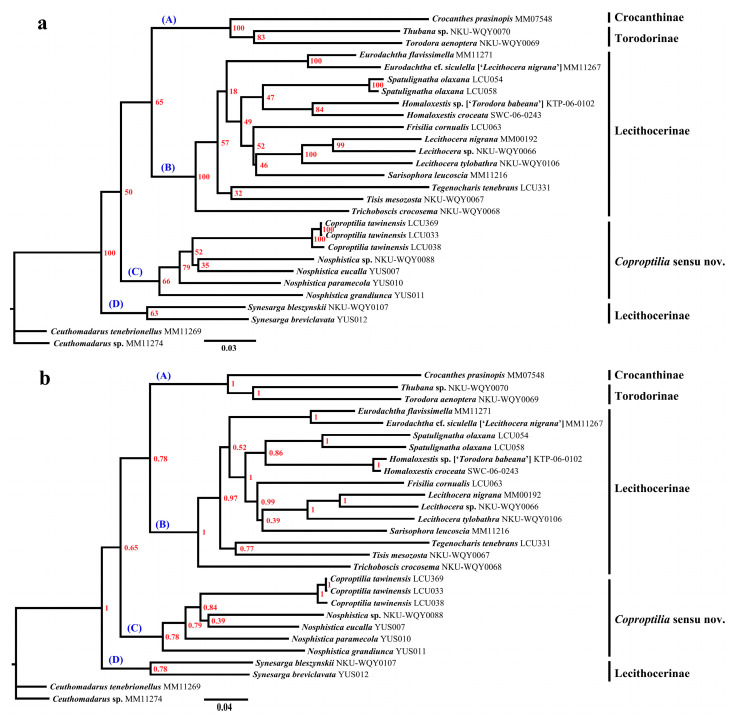
Phylogenetic tree of Lecithoceridae using 28 Lecithoceridae taxa based on a concatenated dataset of 5350 bp. (**a**) The Maximum Likelihood (ML) tree. The number indicates the bootstrap support value (BS). (**b**) Bayesian Inference (BI) tree. The number indicates posterior probability (PP). (**A**–**D**) indicate the four major clades in the phylogenetic tree.

The Maximum Likelihood (ML) tree and Bayesian Inference (BI) tree were constructed based on 28 exemplars representing 25 Lecithoceridae species, and the topological results presented nearly identical results (Figure 1). According to the phylogenetic tree topology, four major clades are recognized: A, B, C, and D. Clade A contains one species from the subfamily Crocanthinae and two species from the subfamily Torodorinae, with the two subfamilies forming a sister branch relationship (BS = 100%, PP = 1.00). Clade B includes 13 species from nine genera within the subfamily Lecithocerinae. Clade C is composed of one species from the genus *Coproptilia* and four species from the genus *Nosphistica*, with *Coproptilia* embedded within *Nosphistica*. Clade D includes two species of the genus *Synesarga*. This clade is located at the base of the phylogenetic tree and forms a sister relationship with the other three clades (BS = 100%, PP = 1.00). Although our molecular phylogenetic tree did not fully resolve the relationships of three clades (A, B, C), most intra-genus (e.g., *Eurodachtha*, *Homaloxestis*, and *Lecithocera*) and intra-species (e.g., *Spatulignatha olaxana* and *Coproptilia tawinensis*) relationships were strongly supported (BS = 100%, PP = 1.00). Notably, within the three individuals of *Coproptilia tawinensis*, specimen YUS038 is a male from Hunan, while YUS033 and LCU369 are male and female specimens from Guangxi, respectively. While YUS033 and YUS038 exhibit consistent male morphological characteristics, YUS033 and LCU369 display a closer genetic relationship, likely due to their geographic proximity. Consequently, the molecular results confirm the matching of males and females for this species with high confidence.

### 3.2. Morphological Results

*Coproptilia* Snellen, 1903.

*Coproptilia* Snellen, 1903: 32 [7]. Type species: *Coproptilia glebicolorella* Snellen, 1903.

*Nosphistica* Meyrick, 1911: 733 syn. nov. [11]. Type species: *Nosphistica erratica* Meyrick, 1911.

*Philoptila* Meyrick, 1918: 111 syn. nov. [28]. Type species: *Philoptila effrenata* Meyrick, 1918.

#### 3.2.1. *Coproptilia tawiensis* Park, 2009

**Figure 2:**
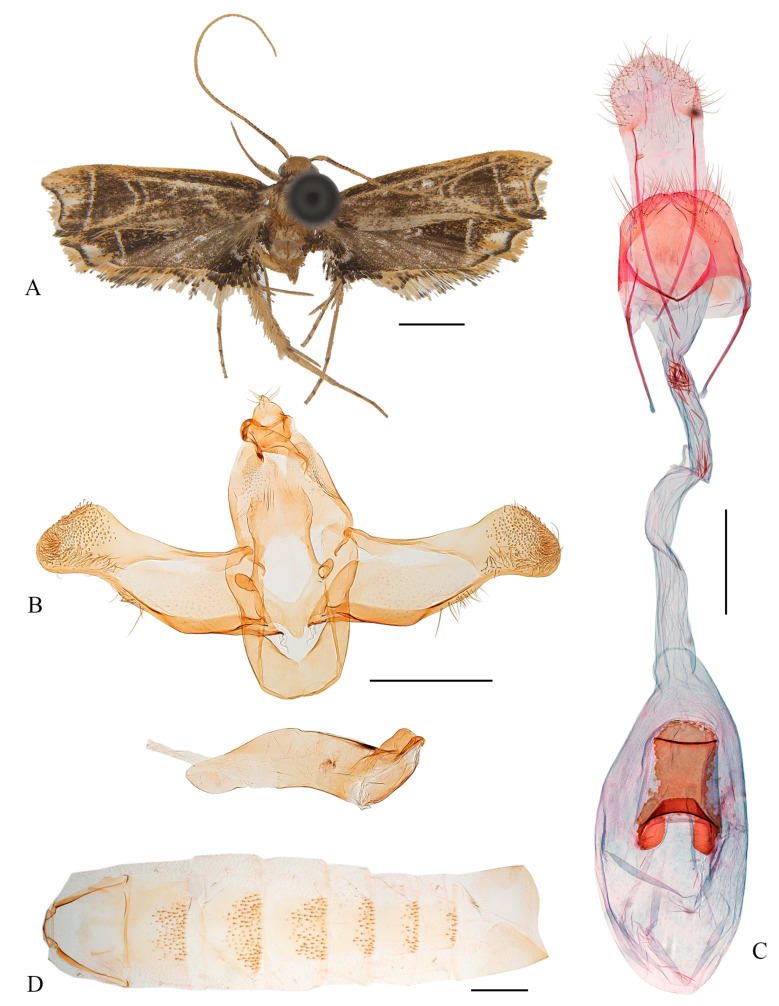
*Coproptilia tawiensis* Park, 2009: (**A**) external features; (**B**) male genitalia; (**C**) female genitalia; (**D**) abdomen. Scales: A = 2.0 mm; B–D = 0.5 mm.

*Coproptilia tawiensis* Park, 2009: 241. Type locality: Philippines (Figure 2).

Material examined: China: 2♂♂, 1♀, Guangxi Prov., Lingui County, Huaping, 842 m, 4 August 2022, leg. H. Sun et al., slide Nos. YUS032♂, YUS033♂, and WLCU369♀, deposited in NKU; 2♂♂, Guangxi Prov., Lingui County, Huaping, 789, 3 August 2022, leg. H. Sun et al., slide No. LCU046, deposited in NKU; 1♂, Guangxi Prov., Lingui County, Huaping, 801 m, 2 August 2022, leg. H. Sun et al., slide No. YUS130, deposited in NKU; 1♂, Hunan Prov., Yizhang County, Mt. Mang, 730 m, 27 July 2020, leg. H. Sun et al. leg., slide No. YUS038, deposited in NKU; 1♂, Yunnan Prov., Pu’er City, Taiyanghe, 1450 m, July 2023, leg. K.J. Teng, slide No. YUS076, deposited in LCU.

Female genitalia (Figure 2C): Abdominal sternite VIII blunt on posterior margin. Apophyses anteriores about 2/3 the length of apophyses posteriores. Ostium bursae large and rounded. Antrum membranous are funnel shaped. Ductus bursae narrow, nearly as long as corpus bursae; ductus seminalis slender, arising from posterior 1/3 of ductus bursae. Corpus bursae elliptical; signum nearly half length of corpus bursae, anterior part semicircular and heavily sclerotized, posterior part rectangular.

Distribution. China (Guangxi, Hunan, Yunnan, new record) and the Philippines.

Note: The female of the species is described for the first time.

#### 3.2.2. *Coproptilia uniformis* Yu, sp. nov. (Figure 3)

ZooBank registration: The LSID for *Coproptilia uniformis* Yu, sp. n. is urn: lsid: zoobank.org: act: AF9EB8A2-3789-4375-AB79-B0876B0FA3C1.

**Figure 3:**
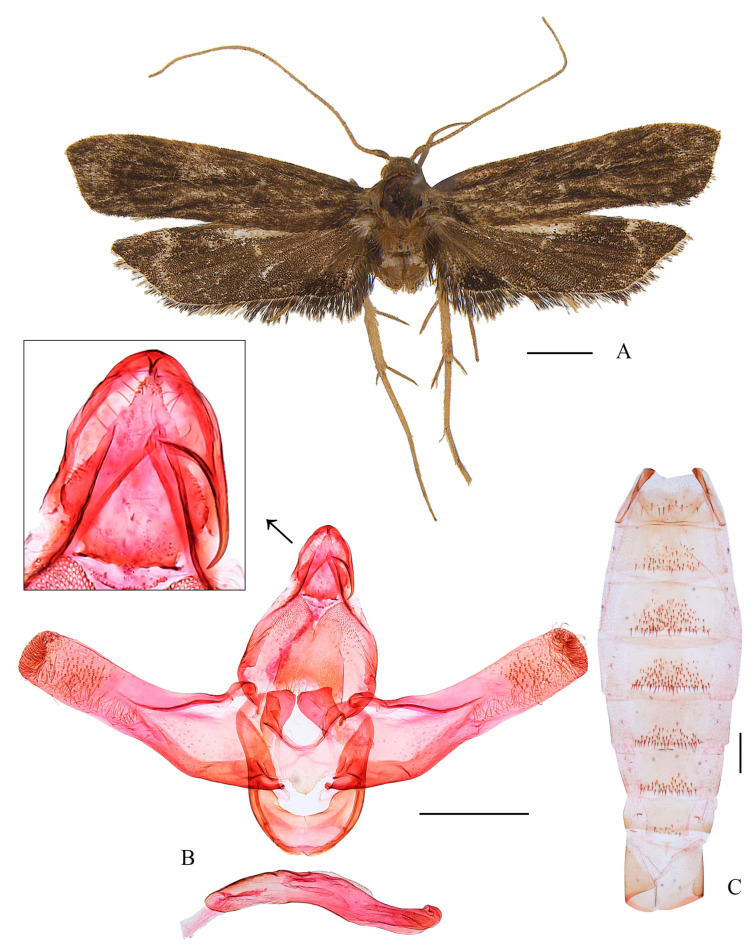
*Coproptilia uniformis* Yu, sp. nov.: (**A**) external features; (**B**) male genitalia; (**C**) abdomen. Scales: A = 2.0 mm; B, C = 0.5 mm.

Material examined: Holotype: ♂, China, Sichuan Prov., Baoxing County, Fengtongzhai, 30.57° N, 102.88° E, 1565 m, 3 August 2016, leg. Y. Fei, slide No. LCU214, deposited in LCU.

Diagnosis: The new species is externally similar to *Nosphistica paramecola* (Wu, 1996), but it can be distinguished by the uncus triangular on the posterior margin, the posterolateral lobe of the juxta widened distally, and the sacculus arched ventrally. *Nosphistica paramecola* has an uncus posteriorly blunt with a semiovate lobe and has widely arm-shaped posterolateral lobes of the juxta, as well as the sacculus nearly straight.

Adult (Figure 3A): Wingspan 20.5 mm. Head dark brown. Antenna yellow, dark brown basally. Labial palpus with second palpomere thickened, third palpomere slender, as long as the second. Forewing with costal margin nearly straight, slightly curved downward distally, apex blunt, termen oblique; ground color dark brown. Hindwing trapezoidal; dark brown, with a subterminal line running from distal 1/4 of costal margin sinuate to before tornus; area between 3A and dorsum black.

Male genitalia (Figure 3B). Uncus pentagonal, triangular on posterior margin. Gnathos with basal plate rounded on posterior margin; median process arched, wide at base, narrowed to middle, thereafter slender to pointed apex. Valva wide at base, narrowed slightly to middle; distal half straight and parallel sided, apex blunt; costal bar free basally; sacculus uniformly wide, arched ventrally. Juxta wide, concave widely on posterior margin, with a semi-ovate extension at middle on anterior margin; posterolateral lobe stout, widened distally, with a nearly straight apical margin. Vinculum U shaped; saccus region reduced. Aedeagus uniformly narrow, more or less S shaped; cornute absent.

Female unknown.

Distribution: China (Sichuan).

Etymology: The specific epithet is derived from the Latin uniformis, referring to the uniformly wide phallus.

#### 3.2.3. *Coproptilia funiuensis* Yu, sp. nov. (Figure 4)

ZooBank registration: The LSID for *Coproptilia funiuensis* Yu, sp. n. is urn: lsid: zoobank.org: act: DAF2AF87-BBF9-445B-9BFA-333079EF5EC9.

**Figure 4:**
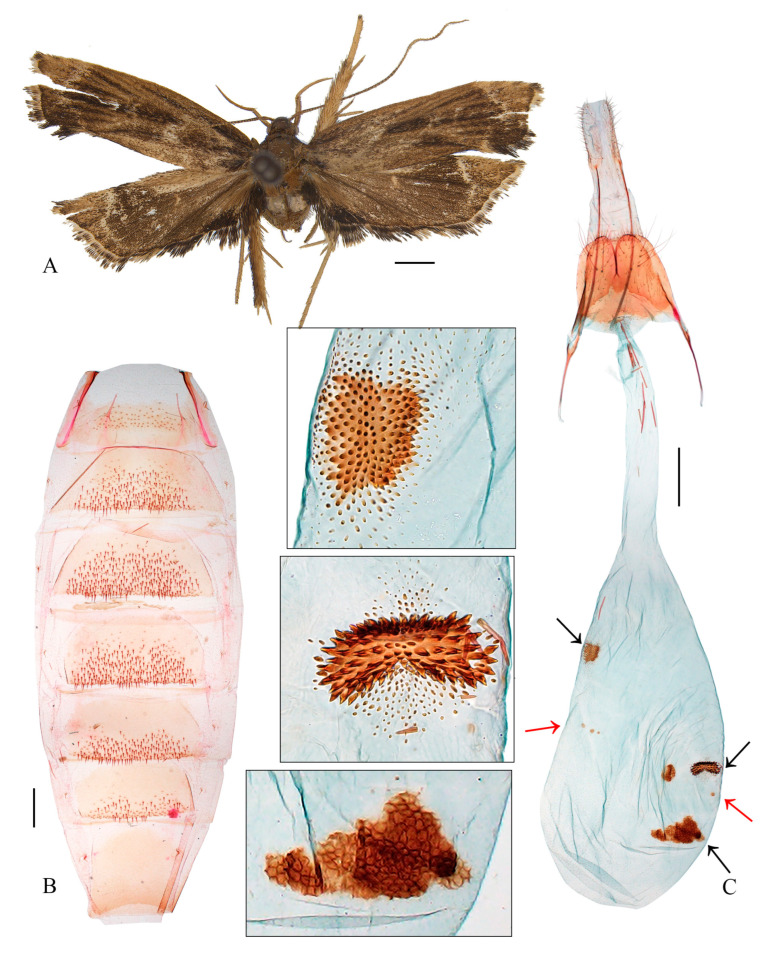
*Coproptilia funiuensis* Park, 2009: (**A**) external features; (**B**) abdomen; (**C**) female genitalia. Scales: A = 2.0 mm; B, C = 0.5 mm. The arrows indicate the signa, among the red arrows are used to label the inconspicuous signa.

Material examined: Holotype: ♀, China, Henan Prov., Mt. Fu’niu, 33.61° N, 111.68° E, 1236 m, 22 July 2023, leg. M.J. Qi and Y.T. Fu, genitalia slide No. LCU373, deposited in LCU.

Diagnosis: The new species can be easily identified by the large size with a wingspan of 26.0 mm and the diversified signa. It is externally similar to *Coproptilia orientana* (Park, 2005) comb. nov., but it can be distinguished by the female genitalia with eight signa varied in shape, and the eighth abdomen sternite is shallowly concave on the anterior margin; *Coproptilia orientana* has two signa that are rounded or elliptical and has an eighth abdomen sternite anteriorly deeply concaved in a U shape.

Adult (Figure 4A): Wingspan 26.0 mm. Head dark brown. Antenna dark brown, paler toward apex. Labial palpus yellowish brown, second palpomere thickened, third palpomere slender, as long as the second. Forewing with costal margin slightly arched, apex bluntly rounded, termen shallowly concave medially; ground color dark brown except brownish yellow on apical area; terminal line pale yellow, arising from distal 1/4 of costal margin to distal 1/5 of dorsum, arched outward medially. Hindwing trapezoidal; ground color dark brown except brownish yellow on apical area; terminal line pale yellow, arising from distal 1/4 of costal margin to before tornus; area between 3A and dorsum black.

Female genitalia (Figure 4C): Abdominal sternite VIII shallowly concave on anterior margin, deeply incised at middle on posterior margin forming two lateral lobes. Apophyses anteriores slightly shorter than apophyses posteriores, with a furcation at middle. Ductus bursae narrowed posteriorly, shorter than corpus bursae; ductus seminalis slender, arising from about posterior 1/5 of ductus bursae. Corpus bursae ovate; with eight signa: two large, dentate, two plate shaped, as well as four small discs.

Male unknown.

Distribution: China (Henan).

Etymology: The specific epithet is derived from the type locality.

## 4. Discussion

**Figure 5:**
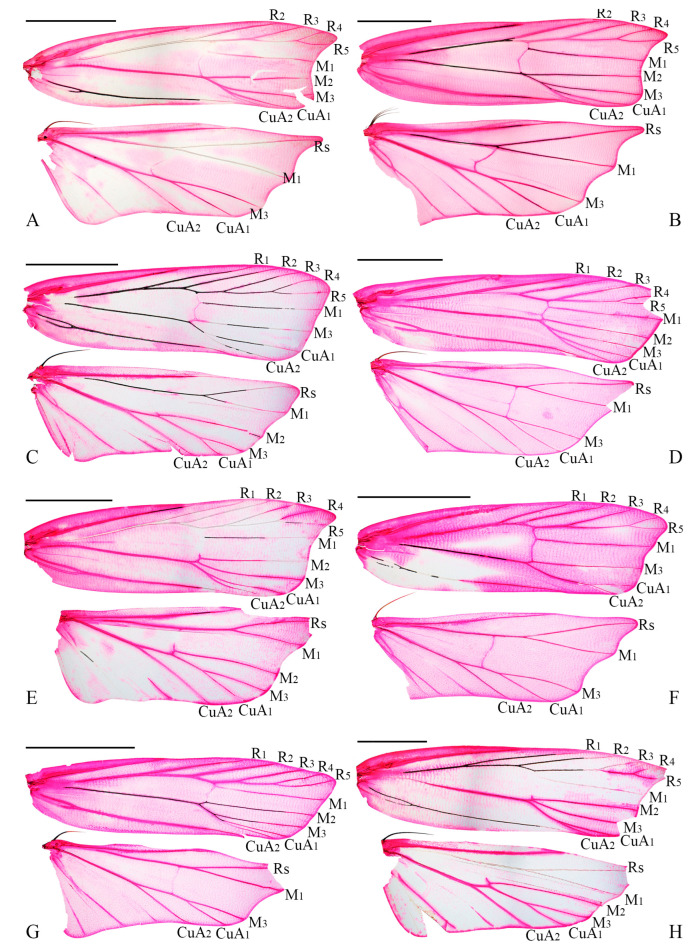
(**A**,**B**) Wing venation of *Coproptilia tawiensis*: (**A**) male, slide No. WLCU373; (**B**) female, slide No. WLCU369. (**C**–**H**) Wing venation of *Nosphistica* spp.: (**C**) *Nosphistica paramecola*, slide No. WLCU213; (**D**) *Nosphistica fenestrata*, slide No. WLCU044; (**E**) *Nosphistica metalychna*, slide No. WLCU045; (**F**) *Nosphistica bisinuata*, slide No. YUS036; (**G**) *Nosphistica fusoidea*, slide No. WLCU368; (**H**) *Nosphistica orientana*, slide No. YUS034. Scales: 2.0 mm.

Wing venation has traditionally been regarded as one of the key characters for defining genera in Lecithoceridae, with particular emphasis on features such as the presence of R_1_ and M_2_ in the forewing and M_2_ in the hindwing [3,9,10,29]. In some cases, it has even been used as the sole character to distinguish genera. For instance, *Torodora* from *Deltoplastis*, *Halolaguna* from *Antiochtha*, and *Lecithocera* from *Sarisophora* are separated based on the presence or absence of M_2_ in the hindwing. Similarly, in this study, *Coproptilia* was distinguished from *Nosphistica* based on the presence of R_1_ in the forewing. However, such classifications often rely on the researcher’s experience and subjective judgment. Moreover, substantial interspecific variation in wing venation is frequently observed within certain Lecithoceridae genera. For example, *Nosphistica* exhibits notable variability in wing venation (Figure 5). *N. metalychna* and *N. fusoidea* possess M_2_ in both the forewing and hindwing; *N. bisinuata* lacks M_2_ in both the forewing and hindwing; *N. fenestrata* and *N. fusoidea* have M_2_ in the forewing but lack it in the hindwing; and *N. paramecola* lacks M_2_ in the forewing but has it in the hindwing. Given these inconsistencies, using the presence of R_1_ in the forewing as the sole criterion to distinguish *Coproptilia* from *Nosphistica* appears unreliable.

The phylogenetic analysis conducted in this study includes three exemplars of *Coproptilia tawiensis* and four exemplars representing four *Nosphistica* species. The topological results of both the Maximum Likelihood (ML) and Bayesian Inference (BI) trees reveal that the *Coproptilia* branch is embedded within the *Nosphistica* branch. This strongly supports the conclusion that *Coproptilia* and *Nosphistica* should be regarded as a single genus. Consequently, we propose synonymizing *Nosphistica* Meyrick with *Coproptilia* Snellen.

Currently, *Coproptilia* Snellen has not been assigned to any subfamily, while *Nosphistica* Meyrick is classified under the subfamily Lecithocerinae [3,10,13]. However, Park suggested that *Nosphistica* might represent an intermediate group between Lecithocerinae and Torodorinae [12]. Our phylogenetic analysis suggests that *Coproptilia* sensu nov. may belong to an unrecognized or yet-to-be-defined subfamily. This conclusion is supported by the placement of clade C, which is distinct from clades A and B in the phylogenetic tree. Morphological evidence further supports this hypothesis. For instance, *Coproptilia* sensu nov. exhibits a polygonal uncus (typically posteriorly bilobed in Lecithocerinae and thorn like in Torodorinae) and a partially free costal bar in the male genitalia (completely free in Lecithocerinae and absent in Torodorinae). These findings suggest that *Coproptilia* sensu nov. is distantly related to other genera within Lecithocerinae. The current scarcity of representative species and the limited availability of molecular data contribute to instability in phylogenetic tree interpretation. Therefore, additional evidence is required to formally establish it as a valid subfamily.

In addition, the genus *Synesarga* forms a distinct branch located at the base of the phylogenetic tree and exhibits a sister relationship with the other subfamilies (Crocanthinae, Torodorinae, Lecithocerinae, and *Coproptilia* sensu nov.). This suggests that the genus *Synesarga* may belong to another independent subfamily or that further research is needed to determine whether it should be classified within the subfamily Lecithocerinae.

## 5. Conclusions

In conclusion, we have revised *Nosphistica* Meyrick as a synonym of *Coproptilia* Snellen based on molecular and morphological evidence and described two new species as well as a new record of *Coproptilia* from China. To better understand the phylogenetic relationships and subfamily taxonomic affiliations within Lecithocerinae, it is crucial to increase sampling and acquire more molecular data.

## Figures and Tables

**Table 1:** Designed primers used in this study.

Gene Region	Forward Primer (5′ to 3′)		Reverse Primer (5′ to 3′)	
Elongation factor 1 alpha (EF-1α)	EF-1α-F	CCYGCCAAYATCACCACTGAAG	EF-1α-R	AGAGGHGGGAACTCYTGGAAGGA
Glyceraldehyde-3-phosphate dehydrogenase (GAPDH)	GAPDH-F	TCACTTGGAVGGTGGHGCCAAGAA	GAPDH-R	AGAGAGATACCAGCDGCAGCATC
Carbamoyl phosphate synthetase domain protein (CAD)	CAD-F	AGTTTRGACTACTGTGTAGTTAAAATA	CAD-R	TGATAAAATAACGCCATCAGGA
Cytosolic malate dehydrogenase (MDH)	MDH-F	TGTTGTCATGGAGCTTGCAGATT	MDH-R	CCCATATAACAACATTCTTWACATCC
Ribosomal protein S5 (RpS5)	RpS5-F	GCAGCATGGCCGTCGATAACAT	RpS5-R	TTGATGAACCCTTGGCAGCATTAAT
Wingless	wingless-F	TGCACAGTGAAAACTTGCTGGAT	wingless-R	GTTACACCTTTCCACAACGAACATG

## Data Availability

All the sequences used in this study were accessed through the GenBank database, and the accession numbers are listed in Table S1. Morphological specimens were deposited at the Insect Collection of Nankai University (NKU), Tianjin, China (NKU), and at Liaocheng University (LCU), Liaocheng, China.

## Supplementary Materials

The following supporting information can be downloaded at https://www.mdpi.com/article/10.3390/ani15030426/s1. Table S1: List of specimen information used for this study.
